# Preliminary study on early diagnosis of Alzheimer’s disease in APP/PS1 transgenic mice using multimodal magnetic resonance imaging

**DOI:** 10.3389/fnagi.2024.1326394

**Published:** 2024-02-14

**Authors:** Meng Xu, Jipeng Liu, Qingguo Liu, Yu Gong, Yinyin Li, Jing Zhang, Shufeng Shi, Yuanyuan Shi

**Affiliations:** ^1^Department of Tuina, Beijing University of Chinese Medicine Third Affiliated Hospital, Beijing, China; ^2^School of Life Sciences, Beijing University of Chinese Medicine, Beijing, China; ^3^School of Acupuncture-Moxibustion and Tuina, Beijing University of Chinese Medicine, Beijing, China; ^4^Department Shenzhen Hospital (Longgang), Beijing University of Chinese Medicine, Shenzhen, China; ^5^Shenzhen Research Institute, Beijing University of Chinese Medicine, Shenzhen, China; ^6^Shenzhen Cell Valley Biopharmaceuticals Co., Ltd., Shenzhen, China

**Keywords:** Alzheimer’s disease, APP/PS1 transgenic mice, early diagnosis, sMRI, rs-fMRI, ^1^H-MRS

## Abstract

Alzheimer’s disease (AD) has an insidious onset and lacks clear early diagnostic markers, and by the time overt dementia symptoms appear, the disease is already in the mid-to-late stages. The search for early diagnostic markers of AD may open a critical window for Alzheimer’s treatment and facilitate early intervention to slow the progression of AD. In this study, we aimed to explore the imaging markers for early diagnosis of AD through the combined application of structural magnetic resonance imaging (sMRI), resting-state functional magnetic resonance imaging (rs-fMRI), and ^1^H-magnetic resonance spectroscopy (^1^H-MRS) multimodal magnetic resonance imaging (MRI) techniques at the animal experimental level, with the aim to provide a certain reference for early clinical diagnosis of AD. First, sMRI scans were performed on 4-month-old amyloid beta precursor protein/presenilin 1 (APP/PS1) transgenic AD model mice and wild type mice of the same litter using a 7.0 T animal MRI scanner to analyze the differential brain regions with structural changes in the gray matter of the brain by voxel-based morphometry (VBM). Next, rs-fMRI scans were performed to analyze the differential brain regions between groups for local spontaneous brain activity and functional connectivity (FC) between brain regions. Finally, ^1^H-MRS scans were performed to quantify and analyze intergroup differences in the relative concentrations of different metabolites within regions of interest (cortex and hippocampus). Compared with wild type mice, the volume of the left hippocampus, and right olfactory bulb of APP/PS1 transgenic AD model mice were reduced, the functional activity of the bilateral hippocampus, right piriform cortex and right caudate putamen was reduced, the functional network connectivity of the hippocampus was impaired, and the relative content of N-acetylaspartate (NAA)in the hippocampus was decreased. In addition, this study found that imaging changes in olfactory-related brain regions were closely associated with AD diagnosis, and these findings may provide some reference for the early diagnosis of AD.

## 1 Introduction

Alzheimer’s disease (AD) is a common progressive neurodegenerative disease that severely affects patients’ daily lives through memory loss and cognitive impairment (Palmer, 2011). Statistics from the 2018 survey found that there are about 50 million people with AD globally, which is expected to triple by 2050, with two-thirds of them living in low- and middle-income countries (Lane et al., 2018). AD is the leading cause of dementia, imposes a heavy financial burden on families and society, and is a growing global health problem (Alzheimer’s Association, 2014; Anand et al., 2014; Lane et al., 2018).

The onset of AD is insidious and progressive, with clinical stages including preclinical stage, mild cognitive impairment stage (MCI), and dementia stage (Breijyeh and Karaman, 2020; Alzheimer’s Association, 2023). Previous studies have shown that pathological changes were already present in the brain before the patients developed mild cognitive impairment, positron emission tomography (PET) scans and cerebro-spinal fluid (CSF) analyses showed that the pathological changes started 38 years before the onset of symptoms (Bateman et al., 2012). By the time symptoms of dementia become apparent, the disease is already in the middle or late stages (Hampel et al., 2011), and diagnosis and treatment of AD at the onset of symptoms is of little help because the pathologic changes and cognitive deficits have accelerated to a more advanced stage (Khan et al., 2020). However, when interventions are given early in the course of AD, they can often significantly slow the progression of AD (Gauthier, 2005; Shibuya et al., 2015). Therefore, early diagnosis is essential as it can buy valuable time for early intervention and treatment, thereby delaying the progression of AD (Breijyeh and Karaman, 2020; Livingston et al., 2020). Early diagnosis of AD has always been a hot and challenging research topic in the field of neuroscience. More and more research are starting to search for early diagnostic methods for AD, including cerebrospinal fluid markers and imaging markers (Dubois et al., 2007; Dubois et al., 2014; Khan et al., 2020).

Magnetic resonance imaging (MRI) technology, due to its non-invasive and rapid detection characteristics, and with the development and maturity of multimodal sequences, has become increasingly widely used in the early diagnosis of AD (Chandra et al., 2019). Structural magnetic resonance imaging (sMRI) has found temporal lobe atrophy and changes in entorhinal cortex volume in early AD patients, which may have certain application value in early diagnosis (Varon et al., 2011; Chandra et al., 2019). Rest-state functional magnetic resonance imaging (rs-fMRI) found that early AD patients may have impaired functional connections in the hippocampus compared with normal elderly person (Wang et al., 2013). ^1^H-magnetic resonance spectroscopy (^1^H-MRS) has found that changes in N-acetylaspartate/creatinine (NAA/Cr) and choline/creatinine (Cho/Cr) ratios in the brain of AD patients are related to the severity of the disease (Franczak et al., 2007; Schott et al., 2010).

However, multimodal MRI has mostly focused on human experiments in the past. Although the results were closer to clinical reality, they were often influenced by other diseases or human factors, which may result in certain false positives or false negatives. In addition, AD patients often have several years of disease progression before experiencing cognitive impairment. There are certain difficulties and limitations in finding the onset characteristics of AD in the early and even asymptomatic stages from patients themselves. The transgenic animal model provides a possibility for studying the early pathogenesis of AD. Amyloid beta precursor protein/presenilin 1 (APP/PS1) transgenic mice have been widely used due to their ability to simulate some typical clinical manifestations and pathological characteristics (Li et al., 2016). As the age changes, different clinical stages of AD can be imitated (Webster et al., 2014; Li, 2017). Therefore, animal experiments will have certain advantages in studying imaging biomarkers for early diagnosis. A single MRI technique cannot fully meet the needs of clinical diagnosis of AD. In order to improve the accuracy, sensitivity, and specificity of diagnosis, the combination of multimodal MRI for early diagnosis of AD has received increasing attention. Through literature review, it was found that there is no systematic report on exploring early diagnostic imaging biomarkers for AD through the combination of sMRI, rs-fMRI, and ^1^H-MRS multimodal MRI technology based on APP/PS1 transgenic mice.

Therefore, this study aimed to reveal the changes in early brain structure, function, and metabolism of APP/PS1 transgenic mice through the combined application of multimodal MRI technology of sMRI, rs-fMRI, and ^1^H-MRS, providing animal level basis for searching for imaging biomarkers for early diagnosis of AD. This study may provide some reference for the early clinical diagnosis of AD in the future.

## 2 Materials and methods

### 2.1 Experimental animals and groups

Eight APP/PS1 transgenic mice (Tg, APP_*swe*_/PSEN1_Δ *E*9_) and eight wild type mice (Wt, [D000268] C57BL/6JNju), SPF grade, male, 4 months old. Wild type mice were used as experimental controls. The experimental animals were purchased from Nanjing Biomedical Research Institute of Nanjing University, with animal certificate number (201805149) and license number (SCXK (Su) 2018-0008). The genotype identification of experimental animals was completed by Nanjing Biomedical Research Institute of Nanjing University to ensure that the experimental animals meet the genotype requirements. The experimental animals were raised according to SPF level standards and were free to eat and drink. The feeding temperature was 20–22°C, the humidity was 50∼60%. The lighting mode was alternating 12 and 12 h. All procedures involving laboratory animals were conducted in strict accordance with relevant animal welfare and ethical principles.

### 2.2 Anesthesia and fixation of experimental animals

After 1 week of adaptive feeding of experimental animals, 7.0T animal MRI scanner (PharmaScan 70/16 US, Bruker, Germany) were performed on the animals. APP/PS1 transgenic mice and wild type mice underwent brain MRI scans alternately. Before and during the MRI scan, the mice were anesthetized. First, the rats were injected dexmedetomidine hydrochloride (100 μg/mL) into the interior lateral thigh muscle to prepare them before scanning using a dose of 0.02 mL per 100 g⋅bw. Second, mice were anesthetized with a mixture of 5% isoflurane/95% O_2_ in an organic glass device. Then applyed a mixture of 2% isoflurane/98% O_2_ at the center of the magnetic field to maintain anesthesia and fix them in a prone position. During the experiment, the real-time small animal vital signs monitor (Model 1025, Small Animal Instruments Inc., USA) was used to closely monitor the temperature, respiratory rate, and heart rate of animals to ensure that they were within the normal range.

### 2.3 MRI data acquisition

#### 2.3.1 sMRI scanning

First, the T2-weighted images were acquired by using the T2_TurboRARE sequence with the following parameters: echo time (TE) = 35 ms, repetition time (TR) = 3366 ms, RARE factor = 8, slices = 30, slice thickness = 0.4 mm, field of view (FOV) = 20 mm × 20 mm, matrix = 256 × 256.

#### 2.3.2 rs-fMRI scanning

Subsequently, the rs-fMRI images based on blood oxygen level-dependent (BOLD) were acquired with the T2Star_FID_EPI sequence using the following parameters: TE = 15 ms, TR = 2000 ms, scan duration = 10 min, slices = 30, slice thickness = 0.4 mm, FOV = 20 mm × 20 mm, matrix = 64 × 64.

#### 2.3.3 ^1^H-MRS scanning

Finally, the hippocampus and cortex were selected as regions of interest (ROI) for ^1^H-MRS scanning. The ROI size was set as 1 mm × 1 mm × 1.5 mm. After homogenizing and suppressing water in the selected ROI brain region, PRESS sequence was used for ^1^H-MRS data acquisition. The parameters were setted as follows: TR = 2500 ms, TE = 17 ms, scan duration = 20 min 50 s.

### 2.4 Pre-processing and index calculation of MRI data

#### 2.4.1 Pre-processing and index calculation of sMRI data

We used the voxel-based methodology (VBM) method to analyze the data. We used MRIcron software to convert the raw DICOM data into an analyzable NIFTI format, and examined the T2-weighted structural images of each mouse to ensure that the data was free of motion artifacts. The data pre-processing was completed using the spmmouseIHEP software package (Nie et al., 2019) based on SPM12.^1^ Firstly, after expanding the spatial resolution (voxel size) of each mouse by ten times, a standard brain tissue probability map based on Paxinos space was used to segment the brain structure images of each mouse using the DARTEL algorithm (Mak et al., 2011). The images were divided into gray matter, white matter, and cerebrospinal fluid. Then, using the gray matter probability map in the brain tissue probability map as a reference standard, we normalized the gray matter images of each mouse to Paxinos space. We resliced the spatially standardized image, and the voxel ize of the resliced image was 1.0 mm × 1.0 mm × 1.0 mm (standard after expanding voxel size). We applied the Jacobian determinant to spatially standardized gray matter images to obtain modulated mouse brain gray matter images, enabling them to perform voxel-by-voxel statistical analysis of brain volume. Finally, we used a Gaussian kernel of 3 mm^3^ Full Width at Half-maximum (FWHM) to smooth the gray image after modulation.

#### 2.4.2 Pre-processing and index calculation of rs-fMRI data

Firstly, we used the MRIcron software to convert the raw DICOM data into an analyzable NIFTI format and checked the brain functional images of each mouse to ensure that there were no motion artifacts in the rs-fMRI data. Next, we used the spmmouseIHEP software package (Nie et al., 2013) based on SPM12 to pre-process the data, and used DPABI software (Yan et al., 2016), FC toolkit, and REST software to calculate the indicators. The data pre-processing steps were as follows: (1) Removing the first 20 volumes of brain imaging data from each mouse to eliminate the impact of magnetic field homogenization on brain functional images. (2) Slice timing: using the second layer of each whole brain as the center point of the timeline, in order to eliminate the differences between different slice collections of each mouse’s entire brain. (3) Realign: using the first whole brain of each mouse as a reference standard, a 6-parameter rigid body transformation was used to perform spatial transformation on the remaining images to eliminate small head movements caused by respiration and heartbeat during image acquisition. Performed spatial averaging on the head motion corrected image to generate the corrected average image and prepared for spatial normalization. (4) Normalization: increased the voxel size of each mouse by 10 times; using the BOLD template in spmmouseIHEP as the reference standard and using the average image of each mouse as the source image, in oder to estimate the transformation parameters for individual transformation into Paxinos space; 12-parameter affine transformation and non-linear deformation were used to apply the transformation parameters to all the head motion corrected images (Zhilkin and Alexander, 2004) to eliminate the differences between subjects and complete the spatial standardization of BOLD brain imaging of each mouse; resliced the spatially normalized images, resulting in a voxel size of 1.0 mm × 1.0 mm × 1.0 mm (standard after expanding voxel size); (5) Smooth: using Gaussian kernel of 2 mm^3^ Full Width at Half maximum (FWHM) to perform Gaussian smoothing on spatially normalized data. (6) Removal of the linear trend: removing linear drift of BOLD signals caused by equipment factors during data collection. (7) Filtering: using a bandpass filter of 0.01∼0.08 HZ to perform bandpass filtering on BOLD data after removing linear drift, in order to retaining only physiological related signals. (8) The calculation of amplitude of low frequency fluctuations (ALFF) and fractional amplitude of low frequency fluctuations (fALFF) values required Gaussian smoothing, and the calculation of regional homogeneity (ReHo) values don’t require Gaussian smoothing. (9) Using the hippocampus (left and right) as the seeds, to calculate the Pearson correlation coefficient between other brain regions and the hippocampus voxel-by-voxel, and obtain the functional connectivity map (FC map) of each mouse. The detailed steps include identify ROI, average time series within seed voxel, voxel-by-voxel analysis, correlation between seed voxel and all other voxels, and statistic-analysis.

#### 2.4.3 Pre-processing and index calculation of ^1^H-MRS data

We used Topspin software (v3.1, Bruker Biospin, Germany) to perform Fourier transform on the raw data of the MRS spectrum, and then adjusted its phase and baseline. All adjusted data were imported into NMPSpec software for quantitative analysis of metabolites, including Cho, Cr, NAA, glutamate and glutamine complex (Glx), and myo-inositol (mI). The chemical shift of metabolites in the brain: NAA is 2.02 ppm, Glx is 2.3 ppm, mI is 3.5 ppm, Cr is 3.05 ppm, and Cho is 3.2 ppm. Due to the generally stable content of Cr in the brain, it is commonly used as an internal reference (Liang et al., 2017) to measure the content of other metabolites. We conducted subsequent statistical analysis using the ratios of NAA/Cr, Glx/Cr, mI/Cr, and Cho/Cr.

### 2.5 Statistical analysis

Based on the generalized linear model (GLM), voxel-by-voxel inter group comparisons were performed on smoothed gray matter images and ALFF, fALFF, ReHo, and FC values. The two-sample *t*-test was established, and the difference brain regions were obtained using a significance level *P* < 0.005, uncorrected, and clusters > 5 voxels.

^1^H-MRS data results were expressed in mean ± SD, and SPSS 20.0 statistical software (IBM, Armonk, NY, USA) was used to conduct a two-tailed Student’s *t*-test. If the data was not normal distribution or the variance was uneven, then non-parametric test was used. *P* < 0.05 indicated a statistically significant difference.

## 3 Results

### 3.1 sMRI analysis

**FIGURE 1:**
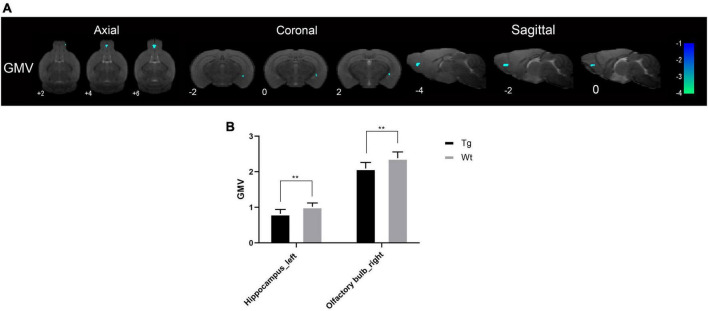
The brain regions with decreased GMV in APP/PS1 transgenic mice compared to wild type mice. **(A)** The differential brain regions were sequentially displayed on axial, coronal, and sagittal sections. The color bar was used to represent the *t*-value (the brighter the color, the higher the *t*-value). **(B)** GMV, gray matter volume; Tg, APP/PS1 transgenic mice; Wt, wild type mice. Data are expressed as mean ± SD (*n* = 8 rats/group). **P < 0.01.

Structural magnetic resonance imaging results showed that the brain regions with reduced gray matter volume (GMV) in APP/PS1 transgenic mice compared to wild type mice were mainly the hippocampus (left) and olfactory bulb (right) (Figure 1). The specific localization information of GMV differential brain regions in brain structural imaging were shown in Supplementary Table 1.

### 3.2 rs-fMRI analysis

#### 3.2.1 ALFF analysis

**FIGURE 2:**
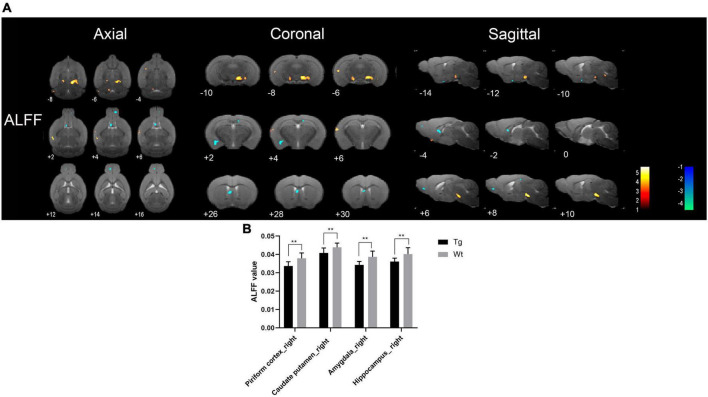
Brain regions with significantly different ALFF value in APP/PS1 transgenic mice compared to wild type mice. **(A)** Warm colors represent brain regions with increased ALFF values, while cool colors indicate brain regions with decreased ALFF values. These differential regions are sequentially displayed on axial, coronal, and sagittal slices. The color bar is employed to denote the *t*-value (brighter colors correspond to higher *t*-values). **(B)** ALFF value of representative brain region. Data are expressed as mean ± SD (*n* = 8/group). **P < 0.01. Tg, APP/PS1 transgenic mice; Wt, wild type mice.

Resting-state functional magnetic resonance imaging results showed that the brain regions with increased ALFF values in APP/PS1 transgenic mice compared to wild type mice mainly included: dentate gyrus (left), hippocampus (bilateral), substantia nigra (bilateral), ventral tegmental area (bilateral), anterior olfactory nucleus (right), locus coeruleus (right), caudate putamen (right), visual cortex (right), insular cortex (right), auditory cortex (right), temporal cortex (right), somatosensory cortex (right);Brain regions with decreased ALFF values in APP/PS1 transgenic mice compared to wild type mice mainly included: amygdala (right), preoptic nucleus (right), piriform cortex (right), hippocampus (bilateral), caudate putamen (right), and septal region (right) (Figure 2). Specific localization information of brain regions with differences in ALFF values on functional brain imaging is shown in Supplementary Table 2.

#### 3.2.2 fALFF analysis

**FIGURE 3:**
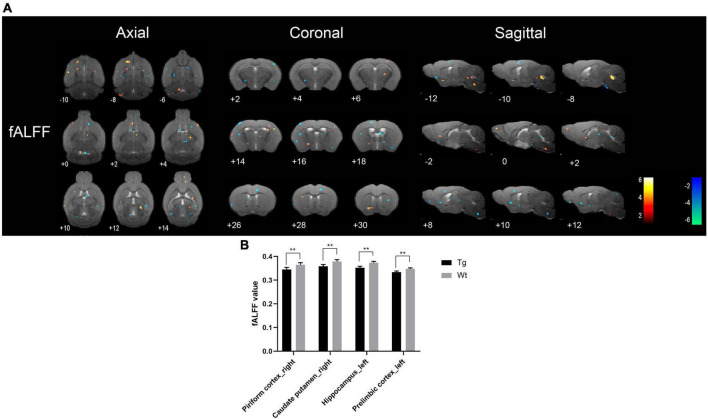
Brain regions with significantly different fALFF value in APP/PS1 transgenic mice compared to wild type mice. **(A)** Warm colors represent brain regions with increased fALFF values, while cool colors indicate brain regions with decreased fALFF values. These differential regions are sequentially displayed on axial, coronal, and sagittal slices. The color bar is employed to denote the *t*-value (brighter colors correspond to higher *t*-values). **(B)** fALFF value of representative brain region. Data are expressed as mean ± SD (*n* = 8/group). **P < 0.01. Tg, APP/PS1 transgenic mice; Wt, wild type mice.

Resting-state functional magnetic resonance imaging results showed that the brain regions with increased fALFF values in APP/PS1 transgenic mice compared to wild type mice mainly included: insula cortex (bilateral), olfactory tubercle (left), piriform cortex (bilateral), entorhinal cortex (right), amygdala (bilateral), caudate putamen (bilateral), accumbens nucleus (right), nucleus ambiguus (right), locus coeruleus (right), septal region (left), auditory cortex (right), ectorhinal cortex (right), somatosensory cortex (bilateral), temporal cortex (right), orbital cortex (right), prelimbic cortex (right), anterior olfactory nucleus (right), hippocampus (left), visual cortex (bilateral), dentate gyrus (left), and olfactory bulb (left); Brain regions with decreased fALFF values in APP/PS1 transgenic mice compared to wild type mice mainly included: hippocampus (bilateral), amygdala (left), piriform cortex (bilateral), caudate putamen (bilateral), accumbens nucleus (bilateral), anterior olfactory nucleus (bilateral), entorhinal cortex (right), caudate putamen (right), insular cortex (right), olfactory bulb (bilateral), septal regions (bilateral), somatosensory cortex (bilateral), visual cortex (bilateral), cingulate cortex (left), motor cortex (bilateral), prelimbic cortex (left), subiculum (bilateral) (Figure 3). Specific localization information of brain regions with differences in fALFF values on functional brain imaging is shown in Supplementary Table 3.

#### 3.2.3 ReHo analysis

**FIGURE 4:**
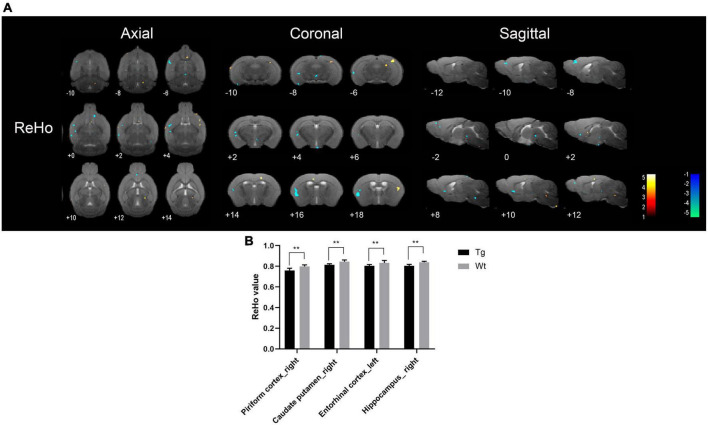
Brain regions with significantly different ReHo value in APP/PS1 transgenic mice compared to wild type mice. **(A)** Warm colors represent brain regions with increased ReHo values, while cool colors indicate brain regions with decreased ReHo values. These differential regions are sequentially displayed on axial, coronal, and sagittal slices. The color bar is employed to denote the *t*-value (brighter colors correspond to higher *t*-values). **(B)** ReHo value of representative brain region. Data are expressed as mean ± SD (*n* = 8/group). **P < 0.01. Tg, APP/PS1 transgenic mice; Wt, wild type mice.

Resting-state functional magnetic resonance imaging results showed that the brain regions with increased ReHo values in APP/PS1 transgenic mice compared to wild type mice mainly included: insular cortex (bilateral), piriform cortex (bilateral), accumbens nucleus (left), olfactory tubercle (left), septal region (left), orbital cortex (right), anterior olfactory nucleus (left), caudate putamen (left), olfactory bulb (left), visual cortex (right), ectorhinal cortex (right), somatosensory cortex (bilateral), auditory cortex (right), temporal cortex (right), visual cortex (bilateral), hippocampus (left), subiculum (left), and motor cortex (bilateral); Brain regions with decreased ReHo values in APP/PS1 transgenic mice compared to wild type mice mainly included: amygdala (right), entorhinal olfactory cortex (bilateral), subiculum (bilateral), accumbens nucleus (right), olfactory tubercle (right), piriform cortex (bilateral), caudate putamen (bilateral), ventral tegmental area (left), insular cortex (right), somatosensory cortex (right), entorhinal cortex (left), ectorhinal cortex (right), hippocampus (bilateral), temporal cortex (right), dentate gyrus (left), orbital cortex (bilateral), auditory cortex (right), olfactory bulb (right), prelimbic cortex (right), and visual cortex (left) (Figure 4). Specific localization information of brain regions with differences in ReHo values in functional brain imaging is shown in Supplementary Table 4.

#### 3.2.4 Key brain regions

Taking the intersection of the discrepant brain regions obtained using ALFF, fALFF, and ReHo analyses, we obtained the common discrepant brain regions, which are often the key brain regions that play an important role in the early stage of AD pathogenesis. The key brain regions with reduced function were the piriform cortex (right), caudate putamen (right), and hippocampus (bilateral).

#### 3.2.5 FC analysis

Based on the results of ALFF, fALFF, and ReHo analyses, the hippocampus was taken as the seed point for FC analysis. rs-fMRI imaging revealed that the brain regions with decreased FC values in APP/PS1 transgenic mice compared to wild type mice mainly included: amygdala (right), hippocampus (right), piriform cortex (bilateral), caudate putamen (bilateral), insular cortex (left), accumbens nucleus (right), anterior olfactory nucleus (right), ectorhinal cortex (right), entorhinal cortex (right), dentate gyrus (right) (Table 1).

**TABLE 1:** The brain regions with decreased FC values in APP/PS1 transgenic mice compared to wild type mice.

Connected region	Brain regions	Voxel size	*T*-value	Peak MNI coordinate (mm)		
				**X**	**Y**	**Z**
	Amygdala	43	−4.03	1.89	5.02	−2.93
	Piriform cortex	9	−3.26	1.79	4.83	2.01
	Insular cortex; piriform cortex	167	−4.11	−2.56	4.26	1.77
Hippocampus	Accumbens nucleus; anterior olfactory nucleus	84	−4.2	0.63	4.32	1.88
	Caudate putamen	138	−5.04	−0.91	4.35	0.36
	Entorhinal cortex;	19	−3.76	3.91	3.32	−4.6
	Ectorhinal cortex					
	Dentate gyrus	57	−3.3	2.95	3.1	−3.43
	Hippocampus	57	−4.32	0.04	1.79	−0.5
	Caudate putamen	31	−3.94	2.17	2.96	0.45

A negative X in the coordinates represents the left brain; a positive X represents the right brain.

### 3.3 ^1^H-MRS analysis

**FIGURE 5:**
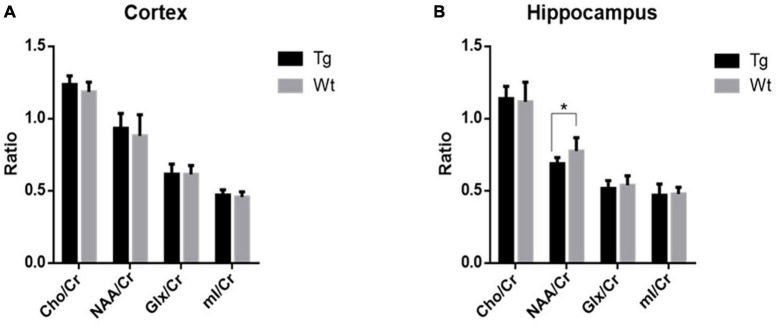
The levels of metabolites within the cortex and hippocampus regions in APP/PS1 transgenic mice (Tg, *n* = 8) compared to wild type mice (Wt, *n* = 8) assessed by ^1^H-MRS. **(A)** Comparative analysis of the relative contents of various metabolites in the cortex. **(B)** Comparative analysis of the relative contents of various metabolites in the hippocampus. Data are expressed as mean ± SD. *P < 0.05.

Based on sMRI and rs-fMRI results, the cortex and hippocampus were taken for ^1^H-MRS analysis. The results revealed that in the cerebral cortex, there were no significant changes observed when comparing APP/PS1 transgenic AD model mice to their wild type control in terms of Cho/Cr, NAA/Cr, Glx/Cr, or mI/Cr ratios. However, within the hippocampus, APP/PS1 transgenic AD model mice exhibited a notable reduction in the NAA/Cr ratio (*P* < 0.05), while the other indicators showed no significant differences (Figure 5).

## 4 Discussion

Researches indicate that AD patients often experience a disease progression period lasting several years before the onset of cognitive impairments (Khan et al., 2020). Because AD patients are often comorbid with other clinical diseases such as hypertension and hyperglycemia (Yu et al., 2020), there are difficulties and limitations in searching for early, or even asymptomatic, pathogenetic features in AD patients from the diagnostic imaging patients themselves. Transgenic animal models provide a potential avenue for studying the early pathogenesis of AD. Among these, the APP/PS1 transgenic AD model mice has gained widespread application due to its ability to effectively mimic some typical clinical manifestations and pathological features (Li et al., 2016; Chen and Zhang, 2022). Previous studies have demonstrated that APP/PS1 double-transgenic AD mice at different ages can replicate various clinical stages of AD development. Based on neurobehavioral assessments and pathological changes, mice at 4 months of age are in a subclinical stage, exhibiting mild Aβ deposition in the brain, while their learning and memory abilities have not yet displayed significant abnormalities (Webster et al., 2014; Li, 2017). Consequently, 4-month-old APP/PS1 transgenic AD model mice can be employed in research related to the early diagnosis of AD.

Despite the advancements in multimodal MRI techniques in AD research, sMRI continues to be an effective adjunct for aiding in clinical early AD diagnosis (Frizzell et al., 2022). sMRI encompasses measurement methods such as VBM and region of interest (ROI) analyses. In this experiment, a morphology-based pixel-wise VBM data processing method was employed, which allows for the detection of subtle structural changes more effectively than other data processing approaches (Wang et al., 2019). sMRI monitors structural brain changes, and in AD, gray matter volume atrophy develops as AD progresses, making it one of the best established imaging biomarkers of AD (Teipel et al., 2006; Bayram et al., 2018). The results of this experiment revealed only slight atrophy in the left hippocampus and the right olfactory bulb. The hippocampus, closely associated with learning and memory (Lisman et al., 2017), plays a pivotal role in the onset and progression of AD (Palmer, 2011; Babcock et al., 2021). Research has indicated that hippocampal atrophy occurs early in AD (Imabayashi et al., 2013; Rao et al., 2022), a finding consistent with the observed reduction in hippocampal volume in the APP/PS1 transgenic AD model mice of this study.

Interestingly, the volume reduction of the olfactory bulb by sMRI was found in this study. In fact, olfactory dysfunction is so prevalent in Alzheimer’s patients that early Alzheimer’s patients exhibit olfactory perceptual deficits that usually occur concurrently with or precede manifestations of classic cognitive deficits such as memory loss (Umeda-Kameyama et al., 2017; Carnemolla et al., 2022). Therefore, one potential approach to the early diagnosis of AD is the detection of olfactory dysfunction (Lafaille-Magnan et al., 2017; MacDonald et al., 2018). This experiment showing reduced hippocampal and olfactory bulb volumes in 4-month-old APP/PS1 transgenic AD model mice. Consequently, subtle structural changes in brain regions such as the hippocampus and olfactory bulb may serve as imaging biomarkers for early AD diagnosis.

Resting-state functional magnetic resonance imaging, owing to its non-invasive and non-destructive nature, coupled with its ability to precisely locate changes in brain functional activity, brings hope to the early diagnosis of AD (Chen et al., 2017). In AD patients, alterations in brain function often precede structural changes (Clark et al., 2012). Consequently, investigating the utility of early stage brain functional changes in the diagnosis of AD holds significant importance. Resting-state fMRI (rs-fMRI) primarily comprises the analysis of brain local spontaneous activity and FC between brain regions (Yousaf et al., 2018). Among these, analyses such as ReHo, ALFF, and fALFF fall under the category of local spontaneous brain activity analysis, reflecting changes in local brain function (Liu et al., 2008; Liang et al., 2014). Increased ALFF, fALFF, and ReHo represent neuronal excitation in localized brain regions, and vice versa indicates neuronal inhibition (Li et al., 2021). FC analysis reflects the functional connectivity between different brain regions (Hutchison et al., 2013). Brain regions in which ALFF, fALFF, and ReHo were all reduced in APP/PS1 transgenic AD model mice compared to wild type mice were mainly the piriform cortex (right), caudate putamen (right), and hippocampus (bilateral), indicating that local neuronal function was impaired in these brain regions. The piriform cortex (PC) is located at the junction of the temporal and frontal lobes and is physiologically involved in olfaction and memory (Steinbart et al., 2023). The caudate putamen and hippocampus are closely related to memory activity (Lisman et al., 2017; González-Franco et al., 2023). Lin et al. (2022) study using rs-fMRI found that neuronal spontaneous activity in the caudate putamen and hippocampus of APP/PS1 transgenic AD model mice was reduced, which is similar to the results of the present study.

Interestingly, the present study also found that APP/PS1 transgenic AD model mice had increased functional activities in some brain regions compared with wild type mice, and the brain regions with increased ALFF, fALFF, and ReHo were mainly the dentate gyrus (left), hippocampus (left), visual cortex (right), insula (right), and temporal cortex (right). The majority of these brain regions are closely associated with cognitive function. Some studies have found widespread abnormalities in neuronal activity in the brains of AD patients, with markedly increased ALFF values in the parahippocampal gyrus and fusiform gyrus in the limbic system, frontoparietal lobe, and brain regions comprising the cerebellar and basal ganglia networks, and suggested that enhanced spontaneous neuronal activity in these brain regions is a neural compensatory mechanism for the cognitive impairment of AD patients (Xiao et al., 2014). Therefore, the synchronized reduction of ALFF, fALFF, and ReHo in piriform cortex (right), caudate putamen (right), and hippocampus (bilateral) could be used as an imaging marker for the early diagnosis of AD, and the compensatory mechanism for the enhanced neuronal activity in some brain regions under pathological conditions also deserves further investigation.

^1^H-MRS stands as a non-invasive detection method capable of segregating signals generated by atomic spins within different chemical environments along the chemical shift axis. It generates spectra consisting of multiple peaks, enabling the quantitative analysis of metabolite content within regions of interest (ROIs) through area calculations (Whitehead et al., 2022). Cr content, which remains relatively stable, is commonly employed as an internal reference to gauge the relative levels of other metabolites (Xing et al., 2020). Notably, NAA reflects neuronal integrity and metabolic status, while Cho, including phosphocholine, phosphatidylcholine, and glycerophosphocholine, mirror cellular membrane integrity. Cr, due to its stability in the brain, is frequently used as an internal reference for assessing the content of other metabolites. MI is associated with the activation or proliferation of glial cells, while Glx is linked to neuronal function and metabolism (Bonavita et al., 1999). The impairment of the hippocampus and cortical regions is closely linked to the onset of AD (Palmer, 2011; Kantarci, 2013). Therefore, this experiment focused on conducting ^1^H-MRS scans in the hippocampus and cortical regions. The results of this study revealed a reduction in NAA levels in the hippocampal region of 4-month-old APP/PS1 transgenic AD model mice compared to their age-matched wild type controls, with no significant changes in other indicators. Decreased NAA levels often indicate neuronal damage, loss, or metabolic inhibition (Hugg et al., 1993). Clinically, reduced NAA markers can be used as predictors of dementia and cognitive impairment (Kantarci, 2013).

Consistent with the results of the present study, Chaney et al. (2021) found reduced NAA content in the hippocampus of APP/PS1 transgenic AD model mice, and similarly, Fowler et al. (2022) found reduced NAA/mI in the hippocampus of APP/PS1 transgenic AD model mice. Multiple studies have shown significant reductions in NAA or NAA/Cr in the hippocampus, posterior cingulate gyrus, parietal cortex, and occipital cortex of AD patients compared to control groups, accompanied by a significant decline in these metabolite changes with the progression of AD clinical course (Ni, 2021). Liang et al. (2017) in their ^1^H-MRS assessment of the hippocampal region in 12-month-old APP/PS1 transgenic AD model mice, observed a decrease in NAA/Cr and Glu/Cr an increase in mI/Cr. These results are consistent with previous reports. Furthermore, the hippocampus is one of the earliest affected regions in AD pathology (Hampel et al., 2011; Rao et al., 2022), the decline in NAA levels in the hippocampal region could potentially serve as a biomarker for early AD diagnosis.

In this study, we found volume atrophy in the left hippocampus using sMRI, reduced spontaneous functional activity of hippocampal neurons bilateral using rs-fMRI, and reduced neuronal metabolism in the hippocampus using ^1^H-MRS, suggesting hippocampal neuronal damage. Using multimodal magnetic resonance techniques, this study demonstrated the important role of the hippocampus in the early imaging diagnosis of AD and also confirmed that neuronal functional changes in brain regions often precede structural morphological changes. FC using the hippocampus as a seed point found that AD mice had impaired functional connectivity between the hippocampus and some brain regions, most of which are closely related to cognitive function, compared with wild mice. Feng et al. (2019) found that the hippocampal functional network was impaired in AD patients using bilateral hippocampus as a seed point. Therefore, we speculate that functional network damage in the hippocampus and other brain regions associated with other cognitive functions in AD patients may be used as an imaging diagnostic indicator.

In addition, we found volumetric atrophy of the right olfactory bulb as well as reduced spontaneous functional activity of neurons in the right piriform cortex, which are two brain regions mainly associated with olfactory function (Brunert and Rothermel, 2021; Schoonover et al., 2021), and they are innervated by a large number of external neuromodulatory inputs (Lu et al., 2019), this result also suggests that there is a close link between olfaction and cognitive function in our AD patients. Linster and Cleland (2016) found that functional connectivity between the olfactory network and the hippocampus may be a key factor in AD progression, and several studies have also confirmed that patients with AD often have olfactory dysfunction, and that olfactory dysfunction is often preceded by cognitive impairment (Dan et al., 2021; Zhang et al., 2022; Pacyna et al., 2023), so the imaging changes in olfactory-related brain regions, such as the olfactory bulb and the piriform cortex, are also important in the early diagnosis of AD, a finding that is also worthy of our further study.

This study has certain limitations. Firstly, it should be noted that this experiment is a preliminary exploratory study. Therefore, it only focuses on 4-month-old APP/PS1 transgenic AD model mice, lacking longitudinal studies and parallel studies of different AD animal models. In future research, we plan to select APP/PS1 transgenic mice and other AD animal models from different age groups for comprehensive investigation and cross validation. Secondly, as a neuroimaging study, this experiment focuses on the macroscopic changes in the brain of AD mice. In future research, we will combine neuroimaging indicators with molecular biology indicators to further improve the accuracy of the results. Finally, this experiment is an animal experiment, although a number of scholars have also used experimental animals to initially explore the biomarkers for early diagnosis of AD, in an effort to provide useful experimental clues for clinical research on AD. However, there is still a gap between these and human studies. In the future research, we will try to explore the early stage of AD and validate the conclusions drawn from this animal experiment, so as to provide some useful clues in the early diagnostic markers of AD.

## 5 Conclusion

In search of early diagnostic imaging markers for AD, we used sMRI, rs-fMRI, and ^1^H-MRS technology to find out: Compared with wild type mice, the volume of the left hippocampus and right olfactory bulb of APP/PS1 transgenic AD model mice was reduced, the functional activity of the bilateral hippocampus, right piriform cortex, and right caudate putamen was reduced, the functional network connectivity of the hippocampus was impaired, and the relative content of NAA in the hippocampus was decreased. In addition, this study found that imaging changes in olfactory-related brain regions were closely associated with AD diagnosis, and these findings may provide some reference for the early diagnosis of AD, but further research is still needed.

## Data availability statement

The original contributions presented in the study are included in the article/Supplementary material, further inquiries can be directed to the corresponding authors.

## Ethics statement

The animal study was approved by the Institutional Animal Care and Use Committee of the Beijing University of Chinese Medicine. The study was conducted in accordance with the local legislation and institutional requirements.

## Author contributions

MX: Writing – original draft. JL: Writing – original draft. QL: Writing – review and editing. YL: Writing – review and editing. YG: Writing – review and editing. JZ: Writing – review and editing. SS: Writing – review and editing. YS: Writing – review and editing.

## Footnotes

1
https://www.fil.ion.ucl.ac.uk/spm/
