# Supplementary material for: Preliminary study on early diagnosis of Alzheimer’s disease in APP/PS1 transgenic mice using multimodal magnetic resonance imaging
Source: Front Aging Neurosci. 2024 Feb 14;16:1326394. doi: 10.3389/fnagi.2024.1326394 (PMC10899441; doi:10.3389/fnagi.2024.1326394)
Supplement: Supplementary file 1 [file Table_1.DOCX]

**Supplementary Table 1** **Specific localization of GMV differential brain regions in structural brain imaging**

|  |  |  |  | **Peak MNI coordinate(mm)** | | |
| --- | --- | --- | --- | --- | --- | --- |
| **Tg vs Wt** | **Brain regions** | **Cluster size** | ***t*-value** | **X** | **Y** | **Z** |
| Tg vs Wt  ↓ | Hippocampus | 25 | -3.24 | -2.75 | 3.79 | -2.23 |
|  | Olfactory bulb | 156 | -3.26 | 0.24 | 3.02 | 3.99 |

Note: GMV: grey matter volume; Tg: APP/PS1 transgenic mice; Wt: wild type mice. A negative X in the coordinates represents the left brain; a positive X represents the right brain. ↓ represents a reduction in GMV。
