# Supplementary material for: Preliminary study on early diagnosis of Alzheimer’s disease in APP/PS1 transgenic mice using multimodal magnetic resonance imaging
Source: Front Aging Neurosci. 2024 Feb 14;16:1326394. doi: 10.3389/fnagi.2024.1326394 (PMC10899441; doi:10.3389/fnagi.2024.1326394)
Supplement: Supplementary file 2 [file Table_2.DOCX]

**Supplementary Table 2 Specific localization of ALFF differential brain regions in functional brain imaging**

|  |  |  |  | **Peak MNI coordinate(mm)** | | |
| --- | --- | --- | --- | --- | --- | --- |
| **Tg vs Wt** | **Brain regions** | **Cluster size** | ***t*-value** | **X** | **Y** | **Z** |
| Tg vs Wt  ↑ | DG; Hippocampus;  Substantia Nigra;  Ventral Tegmental Area | 625 | 5.64 | -0.72 | 4.27 | -3.76 |
|  | Anterior olfactory nucleus | 22 | 3.26 | 0.44 | 5.09 | 2.48 |
|  | Substantia Nigra;  Ventral Tegmental Area | 117 | 3.88 | 1.12 | 4.30 | -2.94 |
|  | Hippocampus | 5 | 3.14 | -2.75 | 4.26 | -2.93 |
|  | locus coeruleus | 40 | 3.48 | 0.82 | 3.73 | -5.41 |
|  | Caudate Putamen | 22 | 3.52 | 2.47 | 3.98 | 0.35 |
|  | Ectorhinal Cortex;  Insular Cortex | 26 | 3.27 | 3.82 | 3.36 | -0.96 |
|  | Auditory Cortex;  Ectorhinal Cortex;  Hippocampus;  Temporal Cortex | 107 | 4.17 | 3.62 | 3.15 | -2.60 |
|  | Auditory Cortex;  Somatosensory Cortex;  Temporal Cortex | 96 | 3.77 | 4.00 | 2.48 | -1.55 |
| Tg vs Wt  ↓ | Amygdala | 14 | -3.42 | 1.80 | 5.47 | -0.81 |
|  | Preoptic Nucleus | 23 | -3.19 | 1.02 | 5.12 | -0.11 |
|  | Amygdala;  Piriform Cortex | 143 | -4.27 | 2.86 | 4.64 | -2.11 |
|  | Hippocampus | 6 | -3.04 | 2.08 | 4.21 | -2.59 |
|  | Caudate Putamen; Septal | 106 | -4.53 | 0.34 | 3.20 | 0.58 |
|  | Hippocampus | 13 | -3.52 | -0.74 | 1.08 | -2.03 |

Note: Tg: APP/PS1 transgenic mice; Wt: wild type mice. A negative X in the coordinates represents the left brain; a positive X represents the right brain. ↑ indicates an increase in the ALFF value; ↓ indicates a decrease in the ALFF value.
