# Supplementary material for: Preliminary study on early diagnosis of Alzheimer’s disease in APP/PS1 transgenic mice using multimodal magnetic resonance imaging
Source: Front Aging Neurosci. 2024 Feb 14;16:1326394. doi: 10.3389/fnagi.2024.1326394 (PMC10899441; doi:10.3389/fnagi.2024.1326394)
Supplement: Supplementary file 3 [file Table_3.DOCX]

**Supplementary Table 3 Specific localization of fALFF differential brain regions in functional brain imaging**

|  |  |  | |  | **Peak MNI coordinate(mm)** | | |
| --- | --- | --- | --- | --- | --- | --- | --- |
| **Tg vs Wt** | **Brain regions** | **Cluster size** | ***t*-value** | | **X** | **Y** | **Z** |
| Tg vs Wt  ↑ | Insular Cortex | 34 | 4.35 | | -0.04 | 5.44 | -6.33 |
|  | Olfactory Tubercle;  Piriform Cortex | 74 | 4.75 | | -1.68 | 5.20 | 2.02 |
|  | Entorhinal Cortex;  Piriform Cortex | 13 | 3.58 | | 3.73 | 4.89 | -2.82 |
|  | Piriform Cortex | 16 | 3.93 | | 2.95 | 5.02 | 0.48 |
|  | Amygdala;  Piriform Cortex | 25 | 5.60 | | 3.24 | 4.38 | -1.29 |
|  | Amygdala;  Caudate Putamen | 15 | 3.64 | | -2.07 | 4.38 | -0.93 |
|  | Accumbens Nucleus;  Caudate Putamen | 90 | 5.64 | | 1.31 | 4.48 | 1.30 |
|  | locus coeruleus | 214 | 5.85 | | 0.82 | 3.42 | -5.30 |
|  | Septal | 20 | 4.19 | | -0.14 | 3.62 | 0.47 |
|  | Insular Cortex | 25 | 3.83 | | 2.27 | 3.69 | 2.11 |
|  | Auditory Cortex;  Ectorhinal Cortex;  Somatosensory Cortex;  Temporal Cortex | 12 | 3.27 | | 3.91 | 3.02 | -1.31 |
|  | Caudate Putamen;  Orbital cortex;  Prelimbic Cortex | 36 | 4.12 | | 0.24 | 3.40 | 1.99 |
|  | Insular Cortex;  Somatosensory Cortex | 44 | 4.36 | | -3.14 | 3.22 | 1.64 |
|  | Caudate Putamen;  Somatosensory Cortex | 6 | 4.26 | | -3.14 | 2.91 | -1.54 |
|  | Somatosensory Cortex | 7 | 3.19 | | 3.91 | 2.65 | -0.61 |
|  | Auditory Cortex | 35 | 3.88 | | 4.10 | 2.20 | -2.62 |
|  | Anterior olfactory nucleus | 5 | 3.40 | | 1.40 | 2.95 | 3.16 |
|  | Caudate Putamen | 8 | 3.40 | | 1.97 | 2.48 | 1.04 |
|  | DG; Hippocampus; | 75 | 5.51 | | -1.51 | 2.06 | -2.96 |
|  | Somatosensory Cortex | 10 | 3.42 | | 2.75 | 2.43 | 1.97 |
|  | RSC; Visual Cortex | 7 | 3.46 | | -2.47 | 1.84 | -4.96 |
|  | Caudate Putamen;  Somatosensory Cortex | 46 | 4.11 | | -1.99 | 1.68 | -0.97 |
|  | Olfactory bulb | 16 | 4.23 | | -0.54 | 2.27 | 3.51 |
|  | Somatosensory Cortex | 6 | 3.22 | | 2.65 | 1.91 | 0.44 |
|  | Somatosensory Cortex | 23 | 4.25 | | -3.05 | 1.82 | -0.73 |
|  | Somatosensory Cortex | 20 | 3.48 | | 1.58 | 1.75 | -0.74 |
|  | Somatosensory Cortex | 11 | 3.35 | | -2.28 | 1.28 | -0.85 |
|  | Visual Cortex | 9 | 3.63 | | 2.26 | 0.77 | -4.40 |
|  | Somatosensory Cortex | 54 | 4.69 | | -2.00 | 0.72 | -1.45 |
| Tg vs Wt  ↓ | Hippocampus | 17 | -3.59 | | 1.60 | 5.07 | -3.75 |
|  | Amygdala;  Piriform Cortex | 19 | -3.52 | | -2.36 | 5.06 | -0.22 |
|  | Piriform Cortex | 6 | -3.43 | | -3.42 | 5.14 | -0.80 |
|  | Caudate Putamen;  Piriform Cortex | 23 | -4.24 | | -2.84 | 4.86 | -0.10 |
|  | Amygdala;  Piriform Cortex | 13 | -3.85 | | -1.78 | 4.60 | -2.22 |
|  | Piriform Cortex | 9 | -3.50 | | 3.34 | 4.60 | 0.47 |
|  | Accumbens Nucleus;  Anterior olfactory nucleus | 10 | -3.45 | | -0.72 | 4.77 | 2.01 |
|  | Amygdala | 6 | -3.32 | | -3.04 | 4.21 | -2.23 |
|  | Hippocampus | 53 | -3.95 | | 1.69 | 4.05 | -1.88 |
|  | Accumbens Nucleus | 9 | -3.51 | | 0.25 | 4.35 | 2.24 |
|  | Entorhinal Cortex | 9 | -3.22 | | 3.72 | 3.73 | -4.71 |
|  | Caudate Putamen;  Insular Cortex | 19 | -3.63 | | 3.24 | 3.91 | -0.59 |
|  | Hippocampus | 9 | -3.32 | | -3.43 | 3.75 | -2.94 |
|  | Anterior olfactory nucleus;  Olfactory bulb;  Piriform Cortex | 27 | -4.02 | | -0.53 | 4.07 | 2.47 |
|  | Insular Cortex | 18 | -4.10 | | 3.43 | 3.89 | 0.70 |
|  | Accumbens Nucleus;  Caudate Putamen | 72 | -6.55 | | -1.01 | 3.72 | 1.76 |
|  | Anterior olfactory nucleus;  Olfactory bulb;  Piriform Cortex | 20 | -3.57 | | 1.11 | 3.87 | 2.94 |
|  | Septal | 34 | -3.79 | | 0.43 | 3.08 | 0.34 |
|  | Caudate Putamen | 19 | -3.79 | | -1.89 | 3.17 | 1.17 |
|  | Caudate Putamen;  Somatosensory Cortex | 40 | -4.06 | | 2.75 | 2.67 | -0.61 |
|  | Septal | 43 | -4.11 | | -0.54 | 2.54 | -0.25 |
|  | Hippocampus | 41 | -5.64 | | -2.66 | 2.09 | -2.72 |
|  | Hippocampus;  Visual Cortex | 6 | -3.21 | | -3.24 | 1.96 | -3.31 |
|  | Cingulate Cortex;  Motor Cortex;  Prelimbic Cortex | 14 | -4.03 | | -0.64 | 1.98 | 2.21 |
|  | RSC;  Subiculum;  Visual Cortex | 83 | -4.44 | | 2.07 | 1.26 | -4.86 |
|  | RSC;  Subiculum;  Visual Cortex | 98 | -4.29 | | -1.70 | 1.18 | -3.80 |
|  | Cingulate Cortex;  Motor Cortex | 48 | -4.61 | | -0.83 | 1.37 | 0.79 |
|  | Somatosensory Cortex;  Visual Cortex | 17 | -4.61 | | -3.06 | 1.10 | -2.15 |
|  | Somatosensory Cortex | 58 | -4.25 | | 2.64 | 0.81 | -0.51 |
|  | Motor Cortex | 43 | -4.24 | | 1.00 | 0.66 | -0.16 |
|  | Prelimbic Cortex | 7 | -4.41 | | -0.16 | 0.98 | 2.79 |
|  | RSC; Visual Cortex | 5 | -3.83 | | -0.84 | 0.36 | -3.45 |

Note: Tg: APP/PS1 transgenic mice; Wt: wild type mice. A negative X in the coordinates represents the left brain; a positive X represents the right brain. ↑ indicates an increase in the fALFF value; ↓ indicates a decrease in the fALFF value.
