# Supplementary material for: Preliminary study on early diagnosis of Alzheimer’s disease in APP/PS1 transgenic mice using multimodal magnetic resonance imaging
Source: Front Aging Neurosci. 2024 Feb 14;16:1326394. doi: 10.3389/fnagi.2024.1326394 (PMC10899441; doi:10.3389/fnagi.2024.1326394)
Supplement: Supplementary file 4 [file Table_4.DOCX]

**Supplementary Table 4 Specific localization of ReHo differential brain regions in functional brain imaging**

|  | |  |  |  | **Peak MNI coordinate(mm)** | | |
| --- | --- | --- | --- | --- | --- | --- | --- |
| **Tg vs Wt** | | **Brain regions** | **Cluster size** | ***t*-value** | **X** | **Y** | **Z** |
| Tg vs Wt  ↑ | | Insular Cortex | 5 | 3.05 | 0.16 | 5.75 | -6.21 |
|  |  | Piriform Cortex | 11 | 3.27 | 2.96 | 5.21 | 0.25 |
|  |  | Accumbens Nucleus;  Olfactory Tubercle;  Piriform Cortex; | 33 | 3.74 | -1.59 | 5.19 | 1.90 |
|  |  | Accumbens Nucleus;  Septal | 19 | 3.56 | -0.43 | 4.29 | 1.18 |
|  |  | Orbital cortex;  Piriform Cortex | 5 | 3.18 | 2.27 | 4.31 | 2.12 |
|  |  | Anterior olfactory nucleus;  Caudate Putamen;  Olfactory bulb;  Piriform Cortex | 28 | 3.89 | -1.69 | 4.10 | 2.71 |
|  |  | Ectorhinal Cortex;  Insular Cortex;  Somatosensory Cortex | 20 | 3.50 | 4.20 | 3.03 | -1.08 |
|  |  | Septal | 11 | 3.32 | -0.24 | 3.42 | 0.58 |
|  |  | Caudate Putamen;  Somatosensory Cortex | 84 | 4.03 | -3.14 | 2.79 | -0.13 |
|  |  | Insular Cortex | 27 | 3.63 | -2.47 | 3.17 | 2.46 |
|  |  | Insular Cortex;  Somatosensory Cortex | 11 | 3.21 | -3.05 | 3.09 | 1.17 |
|  |  | Auditory Cortex;  Temporal Cortex;  Visual Cortex | 27 | 4.01 | 4.10 | 2.44 | -3.79 |
|  |  | DG;  Hippocampus | 20 | 3.92 | -1.60 | 1.95 | -3.08 |
|  |  | Visual Cortex | 21 | 3.49 | -2.48 | 1.25 | -4.50 |
|  |  | Subiculum;  Visual Cortex | 74 | 4.36 | -2.67 | 1.24 | -3.09 |
|  |  | Motor Cortex | 9 | 3.28 | 1.68 | 1.48 | 1.49 |
|  |  | RSC | 12 | 3.91 | 0.42 | 1.05 | -0.63 |
|  |  | Motor Cortex;  Somatosensory Cortex | 16 | 3.88 | -1.13 | 0.96 | -0.74 |
|  |  | Somatosensory Cortex | 13 | 4.05 | -2.19 | 0.83 | -1.45 |
|  |  | Visual Cortex | 6 | 3.32 | 1.77 | 0.60 | -2.40 |
|  |  | Motor Cortex | 27 | 4.07 | 1.48 | 0.83 | 1.02 |
| Tg vs Wt  ↓ | Amygdala;  Entorhinal Cortex;  Subiculum | | 26 | -3.99 | 2.96 | 5.17 | -3.40 |
|  | Accumbens Nucleus;  Olfactory Tubercle;  Piriform Cortex | | 24 | -3.71 | 1.41 | 5.15 | 2.01 |
|  | Caudate Putamen;  Piriform Cortex | | 9 | -3.59 | 2.76 | 4.47 | -0.12 |
|  | Ventral Tegmental Area | | 22 | -3.82 | -0.05 | 3.78 | -3.30 |
|  | Caudate Putamen;  Insular Cortex;  Somatosensory Cortex | | 272 | -5.43 | 3.33 | 3.82 | -0.36 |
|  | Caudate Putamen | | 7 | -3.51 | -2.75 | 3.98 | 0.71 |
|  | Caudate Putamen | | 5 | -3.24 | 2.08 | 4.03 | 0.94 |
|  | Entorhinal Cortex | | 11 | -3.46 | -2.95 | 3.50 | -4.94 |
|  | Amygdala;  Caudate Putamen;  Ectorhinal Cortex;  Hippocampus;  Temporal Cortex | | 57 | -4.51 | 3.14 | 3.40 | -2.01 |
|  | DG; Hippocampus | | 16 | -3.56 | -2.85 | 3.31 | -3.42 |
|  | Caudate Putamen;  Orbital cortex;  Piriform Cortex | | 50 | -4.52 | -1.01 | 3.72 | 1.76 |
|  | Auditory Cortex;  Ectorhinal Cortex;  Hippocampus;  Temporal Cortex | | 34 | -3.80 | 3.91 | 3.12 | -2.96 |
|  | Entorhinal Cortex;  Subiculum | | 7 | -3.22 | -2.85 | 3.11 | -4.59 |
|  | Orbital cortex | | 11 | -3.40 | 0.14 | 2.43 | 2.92 |
|  | Olfactory bulb;  Orbital cortex;  Prelimbic Cortex | | 169 | -4.12 | 0.81 | 1.85 | 3.62 |
|  | RSC;  Visual Cortex | | 9 | -4.03 | -0.74 | 0.26 | -3.34 |

Note: Tg: APP/PS1 transgenic mice; Wt: wild type mice. A negative X in the coordinates represents the left brain; a positive X represents the right brain. ↑ indicates an increase in the ReHo value; ↓ indicates a decrease in the ReHo value.
